# Debonding forces of different pads in a lingual bracket system

**DOI:** 10.1590/2177-6709.22.4.034-040.oar

**Published:** 2017

**Authors:** Valter O. Arima, Mario Vedovello, Heloísa C. Valdrighi, Adriana S. Lucato, Milton Santamaria, Silvia A. S. Vedovello

**Affiliations:** 1 UNIARARAS, Fundação Hermínio Ometto (Araras/SP, Brasil).

**Keywords:** Orthodontic brackets, Dental debonding, Shear strength

## Abstract

**Objective:**

To evaluate the shear bond strength of lingual orthodontic brackets with resin or metal pads, the location of bond failure and the adhesive remnant index (ARI).

**Methods:**

A total of 40 extracted upper premolars were randomly divided into two groups of 20 each: bonding with brackets having (1) pads with extended resin directly on the lingual surface of teeth, and (2) pads with metal custom base on the lingual surface of teeth. The debonding force was measured with an Instron universal testing machine. A Student’s t-test was used to assess the difference between groups (α = 0.05).

**Results:**

The results showed a significant difference between the groups (*p* < 0.001). The shear bond strength of metal pads was significantly higher than resin pads.

**Conclusions:**

Within the limitations of this *in vitro* study, it was concluded that the bond strength of lingual brackets with metal pads was higher than that of brackets with composite resin pads, due to the metal part being a single unit and welded. The failure location in the region between the bracket and the resin pad affected a higher percentage of the resin pads than the metal pads.

## RESUMO

**Objetivo:**

avaliar a resistência de união ao cisalhamento de braquetes ortodônticos linguais com *pads* em resina e metálicos, assim como os locais de ocorrência das falhas, além do Índice de Adesivo Remanescente (ARI).

**Métodos:**

40 pré-molares superiores extraídos foram aleatoriamente divididos em dois grupos de 20 espécimes cada, para a colagem de braquetes com: 1) *pads* em resina, estendida diretamente na face lingual dos dentes; e 2) *pads* com base metálica customizada, na face lingual dos dentes. A força de descolagem dos braquetes foi mensurada por meio de uma máquina de ensaio universal (Instron). O teste *t* de Student foi utilizado para avaliar diferenças intergrupos (α = 0,05).

**Resultados:**

os resultados demonstram haver diferença significativa intergrupos (*p* < 0,001). A resistência de união ao cisalhamento dos *pads* metálicos foi significativamente maior em comparação aos *pads* em resina.

**Conclusões:**

dentro das limitações desse estudo *in vitro*, foi possível concluir que a resistência de união de braquetes linguais com *pads* metálicos foi superior à resistência de braquetes com *pads* em resina composta, uma vez que os *pads* metálicos customizados e os braquetes são fundidos, formando uma única unidade. A porcentagem de falhas de colagem na região entre o braquete e o *pad* foi maior para os *pads* em resina, em comparação aos *pads* metálicos.

## INTRODUCTION

The lingual technique offers significant aesthetic advantages for patients submitted to orthodontic treatment, and therefore demands continuous development of brackets^1^ ^,^ ^2^ and related materials, aiming to obtain results similar to those from conventional labial orthodontic treatment.^3^

For better adaptation to the lingual surface of teeth, compensations have been created at the bracket bases. Also, denominated pads, these compensations are fabricated during the set-up of malocclusion and used to correct torque, angulation and in/out.^4^ However, pads made of composite resin have shown the disadvantage of a high debonding rate, attributed to the fact that the contact area with the lingual surface of the tooth is smaller.^5^

There are several options available for lingual systems, and due to the wide variation in the morphology of tooth surface, making an individual customized base for each bracket has shown to be the most adequate option, with the purpose of avoiding bends in the arches during the course of treatment.^6^ At present, there are systems available in which the lingual bracket is fabricated by means of computer-aided design (CAD) and computer-aided manufacturing (CAM). This technology minimizes the bracket profile and reduces discomfort to the patient, because customized metal pads and brackets are case in a single unit.^7^ In spite of the technology provided by 3D digitization in lingual appliances,^8^ ^,^ ^9^ their cost is still high in comparison to the conventional lingual technique. However, the metal pad fabricated in a personalized manner has been reported to show greater resistance to fracture.^7^ It is worth pointing out that the literature is not conclusive with respect to the resistance of brackets with different bases or pads used in the lingual orthodontic technique.^6^ ^,^ ^10^

The aim of this study was to evaluate the influence of different pads (resin and metal) on the shear bond strength to human enamel,as well as the Adhesive Remnant Index (ARI) and site of failures. The hypothesis tested was that there is difference on the shear bond strength among different pads. 

## MATERIAL AND METHODS

The present study was conducted after being approved by a Research Ethics Committee (988.213/2015).

Sample size was determined in a pilot study (n = 5) using a power analysis of 0.80 and 5% significance level. At least, 12 specimens would have been required to analyze debonding force. In this study, 40 specimens were used to analyze debonding force and the power analysis was found to be 0.90.

The 40 upper premolars were selected from among teeth extracted for orthodontic treatment, through a careful examination, using as exclusion criteria the presence of restorations, cracks or surface defects and previous orthodontic treatment. After that, they were cleaned with periodontal curette (Gracey 7/8, Golgran Millenium^®^, São Caetano do Sul, São Paulo, Brazil), washed with physiological serum, kept in test tubes with thymol 0.1% chlorochemicals (Biotec^®^, Campo Mourão, Paraná, Brazil), and stored in a refrigerator at 4^o^C until they were used. 

**Figure 1:**
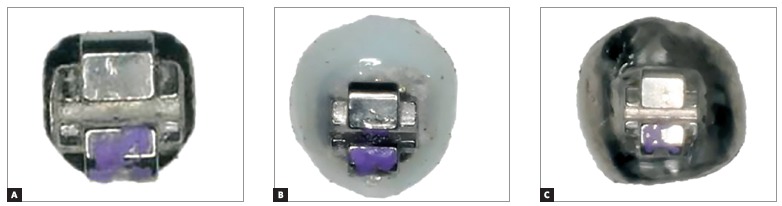
Original bracket (A), bracket with resin pad (B), bracket with a metal pad (C).

The STb™ lingual brackets (Light Lingual System, Ormco^®^, Orange, California, USA) were randomly divided into two groups with 20 each. Group 1 (n= 20) received personalized extended pads made of Transbond XT composite resin (3M Unitek, Monrovia, California, USA) and Group 2 (n = 20), metal pads. The area was standardized by measuring the greatest vertical and horizontal length of each lingual face of all premolars used, by means of the calculation A = π R^2^. Then, the total area (28.66 mm²) average was used as the default (7.06 mm²) (Fig 1). A digital caliper (Starret^®^, São Paulo, São Paulo, Brazil) was used to ensure fidelity.

The teeth were embedded in PVC tubes (Tigre^®^, Santa Catarina, Brazil) measuring 25 mm in diameter by 20 mm high. To fix the teeth, chemically activated acrylic resin Jet (Classic^®^, São Paulo, São Paulo, Brazil) in the sandy phase was used. The lingual surface remained perpendicular to the base of the PVC tube.

The 20 customized pads of Group 1 were prepared by applying a thin layer of Cel Lac (SS White^®^, Rio de Janeiro, Rio de Janeiro, Brazil) insulator on the lingual surface of the teeth, with the use of a disposable paint brush, and drying the region with a light jet of compressed air for 15 seconds. Transbond XT composite resin was used at a 0.5-mm thickness checked with an Iwanson spectrometer (Golgran^®^, São Paulo, São Paulo, Brazil), between the tooth enamel and bracket base, simulating a pad made of composite resin, in which the torque, angulation and in/out were corrected. An extended pad was fabricated around the bracket, covering the lingual surface and crown of the tooth, delimited at approximately 28.66 mm², however not covering the bracket slot region. After this, the resin was light-activated with the FlashMax P3 (Rocky Mountain^®^, Denver, Colorado, USA) appliance for 40 seconds. 

Metal pads were fabricated for Group 2 following the same procedure for isolation of the tooth enamel. In this group, a layer of medium consistency wax was sculpture (Babinete^®^, Curitiba, Paraná, Brazil) and applied with the aid of a Hollenback 3S instrument (Quinelato^®^, Rio Claro, São Paulo, Brazil), covering the lingual surface of the tooth crown, delimiting the same area as that in the previous group. The personalized wax bases were sent for casting in a dental laboratory. The 20 cast parts were polished with a metal polishing disc (Dedeco^®^, New York, New York, USA) and welded to the bracket by means of a laser welding appliance Model LM-D 60 (Sisma^®^, Italy). The weld was calibrated to 0.5mm with Iwanson spectrometer and the diameter of each welding spot was approximately 0.2 mm. All the materials used for joining the weld, orthodontic wire with diameter of 0.020 mm (Morelli^®^, Sorocaba, São Paulo, Brazil), brackets and cast metal parts were made of nickel-chrome. The laser weld filled the space between the customized metal base and bracket, simulating the metal pad on which the torque, angulation and in/out were corrected. It is important to note that torque, in/out and angulation could only be controlled if the orthodontic wire was in position. In this way, all pads were standardized to maintain the size and thickness in relation to the lingual surface.

Before cementing the pads of Groups 1 and 2, they were sandblasted with aluminum oxide particles (50 micrometers) at a distance of 20mm and pressure of 2 bars for 5 seconds. The teeth were washed with water and dried, treated with acetone (Equate^®^, São Paulo, São Paulo, Brazil), aiming at removing residues and creating an area of mechanical retention.

The protocol for fixation of the brackets with metal pads was similar to that of the composite resin pad, with the difference in application of a bonding agent (Metal Primer, Ivoclar, Vivadent^®^, Liechtenstein, Germany) on the entire metal pad. The teeth were cleaned and polished with extra fine pumice stone paste (SS White^®^, Paraná, Brazil) and water, by using a Robson type brush (Microdont^®^, São Paulo, São Paulo, Brazil) for 10 seconds; afterwards washed in running water for 15 seconds and dried with jets of oil- and water-free compressed air. The lingual surfaces were etched with phosphoric acid 37% (Magic Acid Coltene^®^, Rio de Janeiro, Rio de Janeiro, Brazil) for 30 seconds; washed with distilled water for 15 seconds and dried with a jet of compressed air.

During bracket bonding (pads made of resin and metal), a thin layer of Maxcem Elite resin cement (Kerr^®^, Orange, California, USA) was applied on the bases of the pads in the two groups. The brackets were pressed on manually and resin excess was removed with an exploratory probe. Light activation was performed (20 seconds), being 5 seconds for each surface of the bracket, by using a halogen FlashMax P3 (Rocky Mountain^®^, Denver, Colorado, USA) light-activation appliance and light intensity of 4000-6000 mW/cm². The tip of the light-activating appliance was placed as close as possible to the brackets and the specimens were stored in deionized water in an oven (Olidef^®^, São Paulo, Brazil) at 37^o^C, for 24 hours. 

After the storage period had elapsed, the specimens were submitted to the shear bond strength test performed in an universal testing machine (Model 4411; Instron, Canton, MA, USA), at a speed of 1mm/minute^11^. The protocol used for all stages of the study was based on previous literature.^12^

The shear bond strength values were obtained in (N) until failure occurred. The failure location was evaluated and classified as follows: (1) failure between the cement and tooth; (2) failure between the cement and customized pad; (3) failure between the base and pad; (4) failure between the pad and bracket; (5) fracture of the tooth.

The surface of the bracket and tooth were observed under an optical microscope (Olympus Corp, Tokyo, Japan) at 8x magnification. The Adhesive Remnant Index (ARI) was ranked in the sequence, as follows: score 0 = absence of any resin on the enamel surface; score 1 = less than half the resin on the enamel surface; score 2 = more than half the resin on the enamel surface; score 3 = all the resin on the enamel surface, with the impression of the bracket base.^10^

## DATA ANALYSIS 

The shear bond strength values were evaluated as regards the normality by the Shapiro-Wilk test and homoscedasticity of the variances by the Bartlett test. It was verified that distribution was not normal and variances differed. Therefore, the original data were transformed into log and presented normal distribution (*p*= 0.3851) and equal variances (*p*= 0.3291). A Student’s *t*-test was used to assess the difference between groups (α = 0.05).

## RESULTS

**Figure 2:**
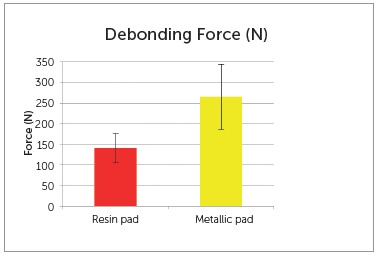
Graphic illustration of the shear bond strength (N) required to remove the lingual orthodontic brackets with different pads.

The results (Table 1) showed significant difference between the brackets (*p*= 0.001). Figure 2 shows the force required to remove the brackets with metal pads was significantly higher than the force to remove the brackets with resin pads.

**Table 1:** Shear bond strength (N) required to remove the lingual orthodontic brackets with different pads.

	Resin pad	Metal pad
Mean (SD)	141.0 (35.4)	265.4 (79.2)
	B	A

Differing letters indicate differences between groups (*p*<0.05).

**Figure 3:**
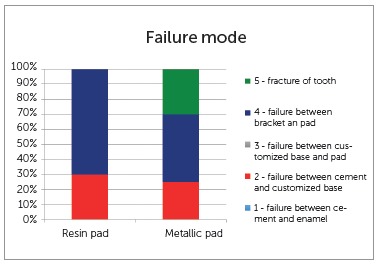
Percentage (%) and failure sites in the experimental groups.

**Figure 4:**
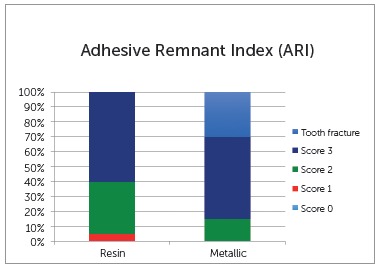
Frequency distribution (%) of Adhesive Remnant Index (ARI) for both groups.

Failure location analysis was performed in the two groups to verify the maximum limit of stress force used for shearing to occur. Failures were observed between the pad and bracket for both groups, with this percentage being 70% for the resin pads, and 45% for the metal pads. There was 30% fracture of the tooth in the customized metal pads, due to the high resistance of force required for shearing (Fig 3). According to Figure 4, there was a higher percentage of ARI with score 3 in both groups.

## DISCUSSION

This study compared the shear bond strength of lingual brackets with different customized pads (composite resin and metal). In the literature, studies that evaluate the shear bond strength of conventional metal brackets are more frequently conducted in comparison with those on the lingual technique,^14^ ^,^ ^15^ while those that evaluate the bond strength of customized brackets are scarce.^6^

To compensate the great anatomic variability of the lingual surface of teeth, the purpose of the resin-based composite pad included at the base of the bracket is to correct the torque, angulation and in/out. However, on the customized metal pad, the compensations are fabricated on the arches,^7^ differently from the resin pads.

There is no previous study in the literature that has evaluated the shear bond strength of lingual brackets with customized pad in comparison with the metal pad in the lingual orthodontics technique. In the present study, metal pads were fabricated and included by means of welding between the metal pad and bracket, favoring lower laboratory costs. 

The results of this study showed that the lingual bracket with metal pad had higher shear bond strength in comparison with those made of composite resin, and the most fragile point of fracture for both groups was between the pad and bracket. Furthermore, it is pointed out that for the resin pads, this percentage was 70%, and for the metal pads, 45%. This high percentage may be due to various factors. Two probable hypotheses are the mechanical retention provided by the bracket base or the type of resin used in this study. Whereas the failure in the metal pads could result from the welded union preformed during the process of fabricating the pad. The pulsed laser process is performed with superimposition of welding spots around the parts, so that there is contact between the customized pad and bracket. This enables better control of the welding energy,^16^ with the consequence of greater fragility of the parts. When polishing around the weld, this region becomes more regular, and may make it more vulnerable to failures. 

During the tests, 30% of fractures at the customized metal bases were in the tooth, a value that may be related to the high resistance obtained in this group. An increased frequency of fracture may be considered a disadvantage. Clinically, this is unlikely to occur, because the forces are distributed more homogeneously in the oral cavity, and the force (265N) used in this *in vitro* study is unlikely to be released.^17^ This is a limitation of *in vitro* studies. On the other hand, during removal of the brackets, some care must be taken, observing the point of force application and avoiding tensile tensions on the enamel. 

A previous study comparing the debonding force and residual adhesives of three different lingual systems (conventional pad resin, Incognito-metal and extended KommonBase-resin)^6^ showed that all the customized lingual bracket systems withstood the orthodontic and masticatory forces. However, the brackets with extended resin pads require a shear force of 104.35(N); with metal pads, 69.29(N); and with conventional resin pad, 60.83(N). The shear bond strength of the customized metal pad was lower in comparison with that of the other brackets, probably due to the fact that the light does not reach the internal region of the metal. In the present study, dual resin cement Maxcem Elite (Kerr^®^) was used with physical and chemical activation, and the results were higher than those observed in a previous study.^6^ Therefore, in the regions where the light is unable to attain adequate polymerization, this may be complemented by chemical activation, guaranteeing better cement properties.^18^

According to a study reference,^13^ an ideal clinical force is around 40 to 120 N. Bond forces used in this study were acceptable for the bond strength of brackets, although the metal pads showed higher values in comparison with the extended pads made of composite resin. The ARI values indicated that the majority of the failures were score 3 (all the resin on the enamel surface) for both groups, indicating that the resin cement used showed a good bond to both the resin pad to tooth enamel and the metal base to tooth enamel. Score 2 occurred two times more frequently in the resin pads than in the metal pads, while score 1 occurred only in 5% of the resin pads.

The limitations of this research are due to the scarcity of studies that evaluate the shear bond strength in the lingual technique. However, the results of this study may contribute to the choice of the lingual accessory most suitable for orthodontic treatment, which will benefit both clinician and patient, with less possibility of debonding and reduction of orthodontic treatment time. 

## CONCLUSION

Within the limitations of this *in vitro* study, it was concluded that the bond strength of lingual brackets with metal pads was higher than that of brackets with composite resin pads, due to the metal part being a single unit and welded. Therefore, the hypothesis tested was accepted. 
